# MRI for diagnosing uterine sarcoma: a systematic review and meta-analysis

**DOI:** 10.1007/s11604-026-01952-4

**Published:** 2026-02-12

**Authors:** Tsukasa Saida, Takahiro Ueda, Maho Kurihara, Mariko Kumazawa, Fuki Shitano, Shinya Fujii

**Affiliations:** 1 Department of Radiology, Institute of Medicine, University of Tsukuba, 1-1-1 Tennodai, Tsukuba, Ibaraki 305-8575 Japan; 2 Department of Diagnostic Radiology, Fujita Health University School of Medicine, 1-98 Dengakugakubo, Kutsukake-cho, Toyoake, Aichi 470-1192 Japan; 3 Department of Radiology, Keio University School of Medicine, 35 Shinanomachi, Shinjuku-ku, Tokyo, 160-8582 Japan; 4 Department of Radiology, Dokkyo Medical University School of Medicine, 880 Kitakobayashi, Mibu-machi, Tochigi, Shimotsuga-gun 321-0293 Japan; 5 Department of Radiology, Osaka Red Cross Hospital, 5-30 Fudegasakicho, Tennoji-ku, Osaka, 543-8555 Japan; 6 Division of Radiology, Department of Pathophysiological and Therapeutic Science, Faculty of Medicine, Tottori University, 36-1, Nishi-cho, Yonago, Tottori 683-8504 Japan

**Keywords:** Magnetic resonance imaging, Uterus, Leiomyosarcomas, Myoma, Apparent diffusion coefficient

## Abstract

**Purpose:**

To systematically review and meta-analyze the diagnostic accuracy of magnetic resonance imaging in differentiating uterine sarcomas from benign leiomyomas, thereby providing updated evidence for the revision of the Japan Radiological Society Diagnostic Imaging Guidelines.

**Evidence acquisition:**

Following the Preferred Reporting Items for Systematic Reviews and Meta-Analyses of Diagnostic Test Accuracy Studies guidelines, a comprehensive literature search was performed by an independent information specialist to identify studies published between July 2019 and April 2025. Inclusion criteria required studies evaluating MRI (the combination of T2-weighted imaging and at least one additional sequence (diffusion-weighted imaging and/or contrast-enhanced imaging)) in patients with myometrial masses suspicious for sarcoma, with diagnostic performance assessable by 2 × 2 contingency tables. The reference standard was histopathology for malignant lesions, whereas benign diagnoses could be established by histopathology or by clinical/imaging follow-up. Data extraction and Quality Assessment of Diagnostic Accuracy Studies-2 quality assessment were performed independently. Pooled sensitivity and specificity were estimated using a random-effects model, and the hierarchical summary receiver operating characteristic (HSROC) curve was generated to estimate the area under the curve (AUC).

**Evidence synthesis:**

Twelve studies met the inclusion criteria. All were retrospective, and substantial heterogeneity existed in patient selection and MRI protocols. The pooled sensitivity of MRI for diagnosing sarcoma was 84.2% (95% confidence interval (CI), 76.3–89.9%), and the pooled specificity was 89.1% (95% CI, 78.1–94.9%). The HSROC-derived AUC was 0.916 (95% CI, 0.876–0.941), indicating high overall diagnostic accuracy. Considerable statistical heterogeneity was noted (*I*^*2*^ = 66.3% for sensitivity and *I*^*2*^ = 95.8% for specificity).

**Conclusion:**

MRI demonstrates high diagnostic accuracy in differentiating sarcoma from leiomyoma. However, substantial heterogeneity in study design, imaging protocols, and diagnostic criteria limits overall consistency across studies. Standardized MRI protocols and diagnostic criteria are essential to improve diagnostic uniformity and clinical applicability.

## Introduction

Uterine sarcoma is a rare malignancy with clinical and imaging features that sometimes overlap with those of benign leiomyomas, leading to diagnostic uncertainty. Accurate preoperative diagnosis is crucial to avoid unnecessary hysterectomy due to a false-positive finding and to prevent delayed surgical management caused by a false-negative diagnosis.

Magnetic resonance imaging (MRI), with its superior soft-tissue contrast, is a key modality for the differential diagnosis and preoperative evaluation of uterine myometrial masses. The 2021 Diagnostic Imaging Guidelines issued by the Japan Radiological Society (JRS) identified the role of MRI in diagnosing uterine sarcoma as a future research question (FQ) [1]. Several MRI features, such as hyperintensity on T1-weighted imaging (T1WI) indicating hemorrhage, intermediate to high signal intensity on T2-weighted imaging (T2WI), and irregular or ill-defined margins, have been reported as characteristic of sarcomas, particularly leiomyosarcomas (LMS) [2–4]. Subsequently, multiparametric MRI, defined as the combined evaluation of standard MRI sequences (e.g., T1WI, T2WI, diffusion-weighted imaging (DWI) with apparent diffusion coefficient (ADC) maps, and contrast-enhanced imaging including dynamic contrast-enhanced (DCE) imaging and delayed contrast-enhanced T1WI (CE-T1WI)), has been shown to be useful, and its comprehensive application is now widely recommended [5–9].

Several systematic reviews have recently assessed the diagnostic performance of MRI for uterine sarcomas [10–12]. To support the forthcoming revision of the JRS Diagnostic Imaging Guidelines, we performed a systematic review and meta-analysis focusing on studies published from 2019 onward, as the previous 2021 edition [1] covered literature only up to that year. By synthesizing the most recent evidence, this study aims to provide the necessary diagnostic updates for clinical practice.

## Evidence acquisition

This systematic review and meta-analysis was conducted and reported in accordance with the Preferred Reporting Items for Systematic Reviews and Meta-Analyses of Diagnostic Test Accuracy Studies (PRISMA-DTA) guidelines [13], which provide standardized criteria for evaluating studies of diagnostic performance.

### Literature search

A comprehensive literature search was performed by an experienced information specialist from the Japan Library Association in consultation with the study investigators. PubMed was systematically searched to identify eligible studies published between July 1, 2019, and April 30, 2025, a period selected to complement the literature reviewed in the 2021 JRS guidelines. The search strategy combined Medical Subject Headings (MeSH) and text words related to uterine sarcoma and MRI: ((“Uterine Neoplasms/diagnosis“[Mesh] AND “Sarcoma“[Mesh] AND “Magnetic Resonance Imaging“[Mesh]) OR ((uterine[TI] OR uterus[TI] OR endometrioid[TI] OR endometrial[TI] OR cervi*[TI]) AND sarcoma*[TI] AND (MRI[TIAB] OR “Magnetic Resonance Imaging“[TIAB]))) NOT “Case Reports“[PT] AND 2019/07:2025/04[DP] AND ENGLISH[LA]. A manual search of the reference lists of key articles and relevant reviews was also performed by S.F. and T.S. to ensure comprehensive coverage. Title and abstract screening, followed by full-text eligibility assessment, were conducted independently by S.F. and T.S. Any discrepancies were resolved through discussion to reach a consensus.

### Inclusion criteria

Studies were deemed eligible if they met all of the following criteria, based on the participant, index test, and target condition framework: (1) participants were patients with uterine myometrial masses suspected of sarcoma; (2) the index test was MRI, including T2WI and at least one additional sequence (DWI and/or contrast-enhanced imaging); (3) diagnostic performance could be assessed by reporting sensitivity and specificity or by providing sufficient data to reconstruct 2 × 2 contingency tables; and (4) the final diagnosis was determined by histopathology or, when histology was unavailable for benign lesions, by appropriate imaging or clinical follow-up.

### Exclusion criteria

Studies were excluded if they met any of the following criteria: (1) non-original research articles, such as case reports, consensus statement, review articles (including systematic review articles), conference abstracts, or editorials; (2) sample size fewer than ten patients; (3) exclusive use of non-clinical advanced analytic methods, such as radiomics or artificial intelligence (AI), without conventional diagnostic assessment, as these techniques are not yet standardized for routine clinical use; (4) not published in English.

### Data extraction and quality assessment

The following variables were extracted from each study: first author, affiliated institution (s), year of publication, patient recruitment period, study design (prospective or retrospective, consecutive enrollment, or case-control), number of patients included in the diagnostic accuracy analysis, characteristics of myometrial masses (counts, benign vs. malignant, and histologic subtypes), reference standard used for the final diagnosis, and technical aspects of MRI acquisition. For MRI, we recorded the acquired sequences, b-values, and ADC cutoff values. We also documented the image interpretation process, including the number of readers, their experience, and whether readings were performed independently or by consensus.

Data extraction and Quality Assessment of Diagnostic Accuracy Studies-2 (QUADAS-2) quality assessment were independently performed by four authors (T.U., M.K., M.K., and F.S.). T.S. and S.F., who were involved in study selection, reviewed and confirmed the final extractions and domain judgments. Any discrepancies were resolved by discussion among all authors, including T.S. and S.F., to reach a consensus. During the QUADAS-2 assessment, we did not downgrade the risk of bias or applicability for studies whose malignant cohorts included subtypes other than LMS, nor for studies that restricted their benign cohorts to diagnostically challenging leiomyomas.

### Statistical analysis

For each included study, extracted diagnostic data were reconstructed into 2 × 2 contingency tables. True positives (TP) and false negatives (FN) were defined as sarcomas that were correctly or incorrectly identified by MRI, respectively. False positives (FP) and true negatives (TN) were benign tumors (leiomyomas) misclassified as malignant or correctly classified as benign.

Meta-analysis was performed using RevMan (Review Manager, version 9.11.0). Study-level sensitivity and specificity were pooled under random-effects assumptions (inverse-variance weighting on logit-transformed proportions). Results were presented as forest plots with 95% confidence intervals (CIs). RevMan was also used to generate the fitted summary receiver operating characteristic (SROC) curve.

To estimate the area under the curve (AUC) with its 95% CI, the same 2 × 2 contingency data were reanalyzed in R (version 4.5.1) using the mada package based on the Reitsma bivariate random-effects model. The pooled AUC and corresponding 95% CI were derived from the fitted hierarchical summary receiver operating characteristic (HSROC) model.

Heterogeneity was assessed using Cochran’s *Q* and Higgins’ *I²* statistics for both sensitivity and specificity, with statistical significance defined as *p* < 0.05. Publication bias was not formally evaluated because the number of included studies was limited and Deeks’ funnel plot asymmetry test is not implemented in RevMan.

## Evidence synthesis

### Study selection and characteristics

The initial database search yielded 59 records. An additional 10 articles were identified through manual searches of relevant reference lists, resulting in a total of 69 unique titles and abstracts for screening. Following the initial assessment, 39 articles were considered potentially eligible based on predefined inclusion criteria and were selected for full-text review.

**Fig. 1:**
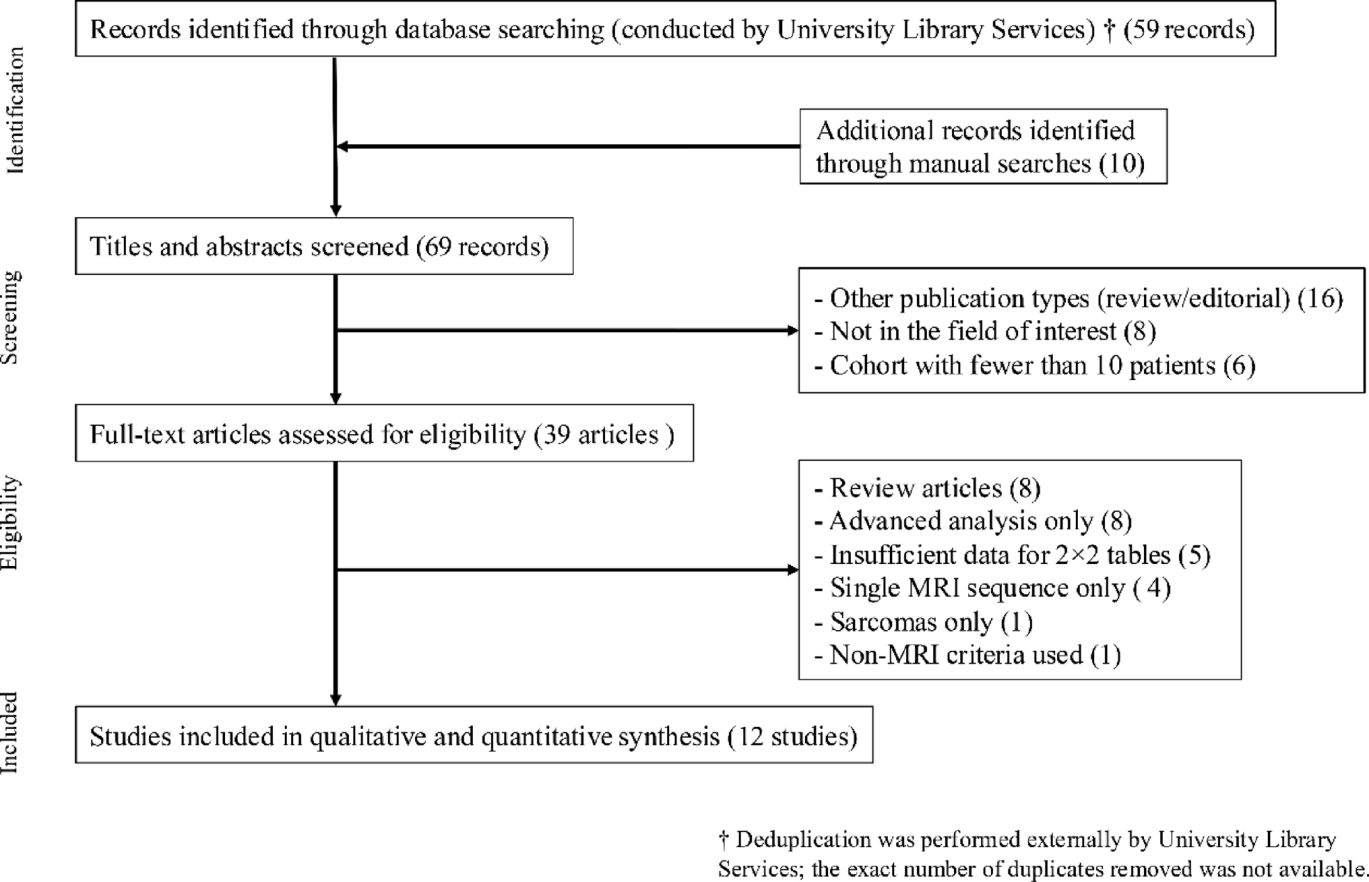
A flow diagram outlining the study selection process

During the full-text screening, 27 studies were excluded for various reasons. The most common reason for exclusion was that the study focused solely on advanced analytic approaches, such as deep learning, machine learning, or radiomics (eight studies), followed by non-original research articles (eight studies). In addition, five studies did not provide sufficient numerical information to reconstruct 2 × 2 contingency tables for meta-analysis. One study included only malignant cases without benign comparators. Four studies assessed only a single MRI sequence, and one study incorporated non-MRI findings into its diagnostic criteria. Consequently, 12 studies were included in both the qualitative and quantitative syntheses. Figure 1 shows the flow diagram of the study selection process.

### Characteristics of included studies

The characteristics of the 12 included MRI studies [14–25] are summarized in Table 1. The number of patients represents those included in the diagnostic accuracy analysis. All studies were retrospective; five were multicenter and seven were single center. Study designs varied, with some investigations using a case control approach enriched with diagnostically challenging leiomyomas, while others enrolled consecutive patients. The malignant cohorts included not only LMS but also smooth muscle tumor of uncertain malignant potential (STUMP), endometrial stromal sarcoma (ESS), carcinosarcoma, adenosarcoma, and other malignant subtypes, reflecting the heterogeneous tumor spectrum described in the original research articles. The benign cohorts often consisted of clinically challenging leiomyomas, such as atypical leiomyomas, T2 hyperintense leiomyomas, or masses measuring 5 cm or larger. Surgical pathology consistently served as the reference standard for malignant lesions, whereas benign tumors were confirmed either histologically or by clinical or imaging follow-up. Regarding MRI protocols, T1WI and contrast enhanced imaging were used in 11 series [14–24], with Ref [25] being the only exception. Ten studies (83%) used DWI [14, 16, 17, 19–25]. Among those using DWI, eight utilized ADC maps [14, 16, 17, 20–24]. The maximum b-values were 800 s/mm^2^ in two studies [14, 22], 1000 s/mm^2^ in four [16, 19, 20, 23], and 500 s/mm^2^ in one [17]. Analytical approaches varied: four studies specified ADC cutoffs (0.905 × 10⁻³ mm²/s [16], 1.187 × 10⁻³ mm²/s [20], 0.839 × 10⁻³ mm²/s [22], and 1.05 × 10⁻³ mm²/s [23]), whereas others used qualitative or semi-quantitative assessment. Reader numbers, experience, and reading paradigms were heterogeneous, consistent with the overall diversity of study designs.

**Table 1:** Characteristics of the included studies

First author, publication year [Ref]	Center	No. of patients analyzed (sarcomas, CS and adenosarcoma))	Comparator group	Sequence
Tong, 2019 [14]	Single	Sarcoma: 11–17 (LMS: 10, ESS:1–7), CS:1–7	Leiomyoma: 1942	T1WI, T2WI, DWI (b = 0, 50, 400, 800), ADC, CE
Xie, 2019 [15]	Single	Sarcoma: 19 (LMS: 10, ESS: 5, Undifferentiated: 3, Rhabdomyosarcoma: 1), CS: 7, Adenosarcoma: 3	Atypical leiomyoma: 49 (Cellular: 8, Degenerated: 23, No degeneration: 18)	T1WI, T2WI, Dynamic CE
Abdel Wahab, 2020 [16]	Multi	Validation set 1 Sarcoma: 14 (LMS: 2, STUMP: 3, ESS:1, Undifferentiated: 7, Rhabdomyosarcoma: 1), CS: 2	Atypical leiomyoma: 26 (Cellular: 1, Degenerated: 8, Lipoleiomyoma: 1, Bizarre:1, No degeneration: 15)	T1WI, T2WI, DWI (b = 0, 1000), ADC (≤ 0.905 × 10^− 3^ mm^2^/s), Dynamic CE, CE
		Training set Sarcoma: 31 (LMS: 17, STUMP: 2, Endometrial stromal nodule: 1, ESS:7, Undifferentiated: 2, Liposarcoma: 2), CS: 19, Adenosarcoma: 1	Atypical leiomyoma: 105 (Cellular: 7, Degenerated: 55, Lipoleiomyoma: 3, Hemorrhagic: 1, Necrobiotic:17, No degeneration: 5, Follow-up at MRI only: 17)	T1WI, T2WI, DWI (b = 500, 800, or 1000), ADC (≤ 0.905 × 10^− 3^ mm^2^/s), Dynamic CE, CE
Aminzadeh, 2021 [17]	Single	Sarcoma: 11 (LMS: 10, STUMP: 1)	Leiomyoma ≥ 5 cm: 32 (Cellular: 1, Symplastic: 2, Infarcted: 7)	T1WI, T2WI, DWI (b = 500), ADC, CE
Jagannathan, 2021[18]	Single	LMS: 17 (MRI predictive score) (total 19)	Leiomyoma: 25 (Atypical: 7, [Infarcted: 2, Hydropic changes: 2, Cellular: 1, Cotyledonoid dissecting: 1 and Rupture: 1])	T1WI, T2WI, CE
Najibi, 2021 [19]	Single	Sarcoma: 37	Leiomyoma: 26	T1WI, T2WI, DWI (b = 1000), Dynamic CE
Matsuura, 2022 [20]	Single	Sarcoma: 32 (LMS: 16, STUMP: 2, ESS: 12, Undifferentiated: 2)	T2 hyper intensity leiomyoma: 34 nodules/30 patients (Cellular: 15, Degenerated: 19)	T1WI, T2WI, DWI (b = 0, 500, 1000), computed DWI (b = 1500–4000), ADC (≤ 1.187 × 10⁻³ mm²/s), CE
Toyohara, 2022 [21]	Multi	Sarcoma: 57 (LMS: 36, STUMP: 3, ESS: 6, Undifferentiated: 10, Spindle-cell: 2), Adenosarcoma: 6	Leiomyoma: 200	T1WI, T2WI, DWI, ADC, Dynamic CE, CE
Wang, 2022 [22]	Single	Sarcoma: 32	Cellular leiomyoma: 51	T1WI, fs-T2WI, DWI (b = 800), ADC (≤ 0.839 × 10⁻³mm²/s), Dynamic CE
Rosa, 2023 [23]	Multi-	Sarcoma: 12 (LMS: 7, STUMP: 1, ESS: 4), Adenosarcoma:1	Leiomyoma: 41	T1WI, T2WI, DWI (b = 50, 800, 1000), ADC (≤ 1.05 × 10⁻³ mm²/s), CE
Al Khuri, 2024 [24]	Multi	Sarcoma: 26 (LMS: 13, STUMP: 13)	Leiomyoma: 218	T1WI, T2WI, DWI, ADC, Dynamic CE
Yamanishi, 2025 [25]	Multi	Sarcoma: 139 (LMS: 80, STUMP: 16, ESS: 31, ESS NOS: 6, Undifferentiated: 6)	Leiomyoma: 141	T2WI, DWI

LMS, leiomyosarcoma; ESS, endometrial stromal sarcoma; CS, carcinosarcoma; STUMP, smooth muscle tumor of uncertain malignant potential; T1WI, T1-weighted imaging; T2WI, T2-weighted imaging; DWI, diffusion-weighted imaging; ADC, apparent diffusion coefficient; CE, contrast-enhanced T1WI

### Quality assessment (QUADAS-2)

As summarized in Table 2, the overall methodological quality of the included studies was moderate to good, with generally low concerns regarding applicability. Most studies showed a low risk of bias in patient selection and index testing, although incomplete reporting, particularly in flow and timing, frequently led to “Unclear” ratings. Case-control sampling in some studies raised minor concerns regarding spectrum representativeness. As prespecified, we did not downgrade studies that included malignant subtypes other than LMS, as these were part of the original sarcoma cohorts. For benign lesions, cohorts frequently included diagnostically challenging leiomyoma subtypes, which were considered appropriate for the clinical context of this review.

**Table 2:** Diagnostic performance metrics

First Author, publication year [Ref]	Risk of bias				Concern of applicability		
	Patient selection	Index test	Reference standard	Flow and timing	Patient selection	Index test	Reference standard
Tong, 2019 [14]	Low	Low	High	Unclear	Low	Low	Low
Xie, 2019 [15]	Low	Low	Low	Unclear	Low	High	Low
Abdel Wahab, 2020 [16]	Low	Low	Low	Unclear	Low	Low	Low
Aminzadeh, 2021 [17]	High	Low	Low	High	Low	Low	Low
Jagannathan, 2021[18]	Low	Low	Low	Unclear	Low	High	Low
Najibi, 2021[19]	High	Low	Low	Unclear	Low	Low	Low
Matsuura, 2022 [20]	Low	Low	Low	Unclear	Low	Low	Low
Toyohara, 2022[21]	High	High	Low	Unclear	Low	Low	Low
Wang, 2022 [22]	Low	Low	Low	Unclear	Low	Low	Low
Rosa, 2023 [23]	Low	Low	High	Unclear	Low	Low	Low
Al Khuri, 2024 [24]	Low	Low	Low	Unclear	Low	Low	Low
Yamanishi, 2025 [25]	High	Low	Low	Unclear	Low	Low	Low

For the index test, concerns were noted regarding variable reader blinding, inconsistent use of prespecified ADC thresholds, and heterogeneous reading paradigms. The main issue in the reference standard domain was differential verification: malignant tumors were pathologically confirmed, whereas some benign lesions were verified only by imaging or clinical follow-up, potentially introducing bias. The risk of bias in the flow and timing domain was often unclear due to unspecified intervals between MRI and the reference standard and was considered high when verification pathways differed. Applicability concerns were generally limited, though they arose in studies using highly site-specific protocols, such as unusual b values, contrast timing, or ROI definitions. Studies with broader recruitment or multicenter designs demonstrated better applicability.

### Diagnostic performance of MRI for diagnosing uterine sarcoma

**Fig. 2:**
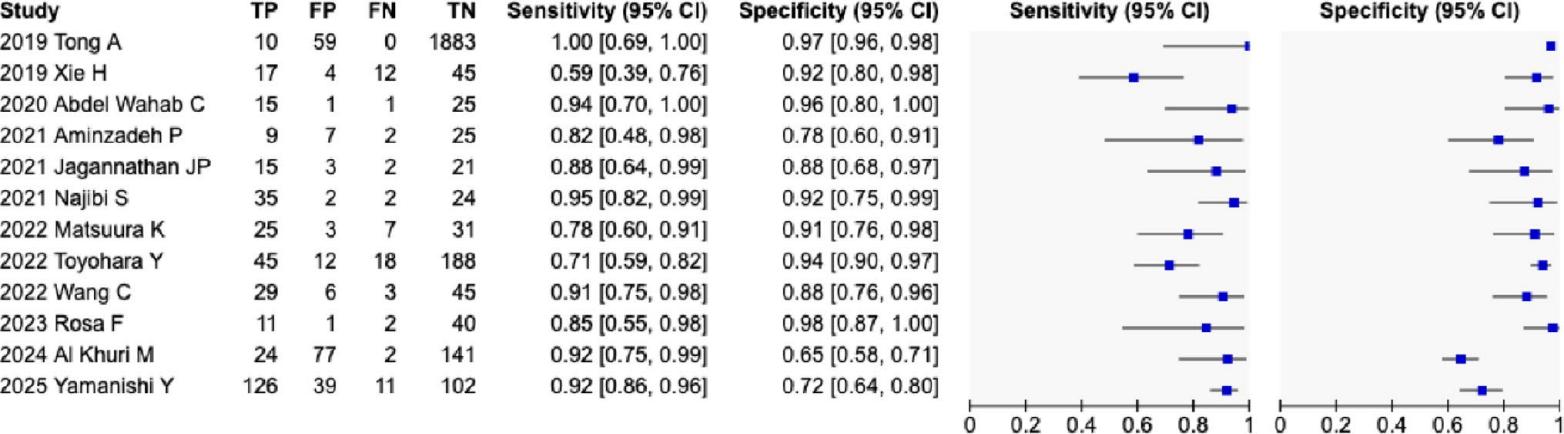
The sensitivity and specificity of each study TP, true positives; FP, false positives; FN, false negatives; TN, true negatives

The sensitivity and specificity of each study are presented in Fig. 2. Random-effects pooling showed a sensitivity of 84.2% (95% CI, 76.3–89.9%) and a specificity of 89.1% (95% CI, 78.1–94.9%).

**Fig. 3:**
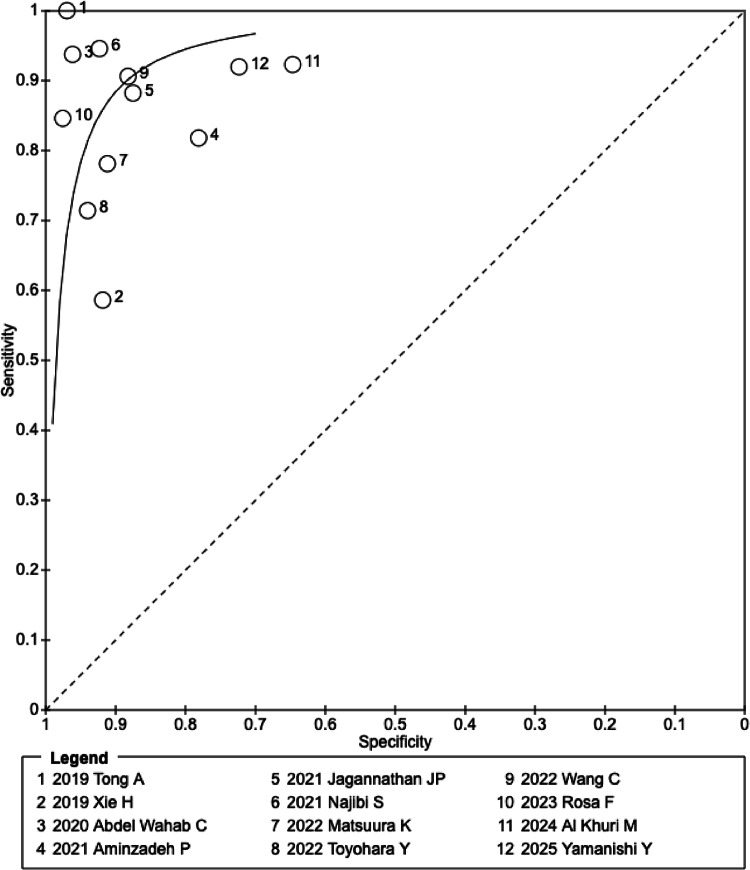
The fitted summary receiver operating characteristic curve

Using the same data, HSROC analysis produced an AUC of 0.916 (95% CI, 0.876–0.941), indicating high overall diagnostic accuracy. The SROC curve is shown in Fig. 3.

Heterogeneity was substantial, with *I²* = 66.3% for sensitivity (*Q* = 32.65, df = 11, *p* = 0.0006) and *I²* = 95.8% for specificity (*Q* = 264.57, df = 11, *p* < 0.001). Heterogeneity was therefore considered significant for both parameters.

## Discussion

This systematic review and meta-analysis of 12 MRI studies, conducted to support the revision of the JRS Diagnostic Imaging Guidelines, confirmed pooled sensitivity and specificity of 84.2% and 89.1%, respectively, with an HSROC AUC of 0.916. These findings support the use of MRI as a robust diagnostic tool for evaluating myometrial masses.

MRI serves as a key modality for differential diagnosis of uterine myometrial masses. Nevertheless, its recommendation for the comprehensive diagnosis of sarcoma is based on evidence from both qualitative features and quantitative ADC values, as most studies involved small sample sizes and institution-specific ADC cutoff values that are not readily generalizable, reflecting the challenges inherent in conducting large-scale studies of this rare tumor. Recently, several systematic reviews have evaluated the diagnostic performance of MRI for uterine sarcomas [10–12]. Hindman et al. [10] reported an overall diagnostic accuracy of 88%–95%, with sensitivity ranging from 83%–100% and specificity from 88%–100%. Raffone et al. [12] demonstrated a pooled sensitivity of 90% (95% CI, 84%–94%), specificity of 96% (95% CI, 96%–97%), positive likelihood ratio of 13.55 (95% CI, 6.20–29.61), negative likelihood ratio of 0.08 (95% CI, 0.02–0.32), diagnostic odds ratio of 175.13 (95% CI, 46.53–659.09), and an AUC of 0.976. These systematic reviews reported substantial heterogeneity in diagnostic accuracy, indicating that performance varies with differences in study populations, reference standards, and MRI feature assessment, similar to what we observed in our review. However, the consistently high specificity underscores the clinical utility of MRI by enabling confident identification of malignancy, which is essential for guiding en bloc resection and avoiding hazardous morcellation.

The present meta-analysis demonstrates that MRI provides high diagnostic accuracy for differentiating uterine sarcomas from leiomyomas, with performance comparable to previous systematic reviews [10–12]. However, a significant challenge in interpreting these results lies in the pathological diversity of the malignant cohort. Most studies primarily targeted LMS, but several also included STUMP and ESS, each differing in biological behavior and MRI appearance. STUMP often shows intermediate features between leiomyoma and LMS, contributing to diagnostic ambiguity [26]. ESS may demonstrate characteristic imaging findings such as low-signal-intensity bands, cystic or necrotic areas, and worm-like nodular extension along the myometrium [27, 28]. Carcinosarcoma and adenosarcoma are no longer classified as true sarcomas but as endometrial epithelial tumors or mixed epithelial and mesenchymal tumors [15, 16]. These lesions typically present as heterogeneous large masses filling the uterine cavity, whereas adenosarcoma more commonly protrudes into the cervical canal with frequent cystic changes [29, 30]. Such pathological diversity likely contributed to variability in diagnostic performance, particularly specificity. Similarly, the control groups often comprised various leiomyoma subtypes, including cellular, bizarre, and degenerative variants. These benign subtypes frequently show heterogeneous T2 signal, irregular margins, hemorrhage, or restricted diffusion, mimicking sarcomatous features and leading to false-positive findings.

Among individual MRI parameters, T2WI provides excellent soft-tissue contrast and plays a key role in the initial assessment of myometrial lesions. The studies included in our meta-analysis evaluated T2WI using various criteria, including margin irregularity or infiltrative patterns [14–18, 20–25], signal intensity grading relative to reference tissues [14, 16–20, 22–25], and specific findings such as internal heterogeneity [17, 18, 20, 23, 24], T2-hypointense bands [20], or endometrial cavity interruption [15]. Generally, homogeneous low signal intensity on T2WI, comparable to that of skeletal muscle, is strongly associated with leiomyomas. In contrast, intermediate to high signal intensity on T2WI relative to the background myometrium has been correlated with malignant lesions such as LMS. However, this finding also overlaps with certain benign variants, including cellular and atypical leiomyomas, thereby limiting its specificity [10]. While an irregular margin was frequently identified as a malignancy indicator in the reviewed studies [14–18, 20–25], its diagnostic weight is often regarded as weak to moderate, partly because the definition of “irregular” has varied among reports. To address this, Hindman et al. [10] adopted the terminology from the American College of Radiology Thyroid Imaging Reporting and Data System, which defines “irregular,” “lobulated,” and “smooth” margins.

T1WI is useful for detecting intratumoral hemorrhage, and the studies evaluated this meta-analysis using various criteria, primarily focusing on high signal intensity relative to reference tissues such as skeletal muscle, myometrium, or bone marrow [14–24]. Specific attention was given to signal distribution, such as distinguishing central patchy foci from a peripheral hemorrhagic rim [23, 24], and this feature was adopted as a classification algorithm in some studies [18, 23]. Despite being traditionally regarded as a potential indicator of malignancy [20, 31, 32], the evidence linking hemorrhage to sarcoma remains limited and inconsistent because T1 high signal intensity is also observed in benign degenerative or infarcted leiomyomas [10], which can lead to diagnostic overlap.

The presence of necrosis is a characteristic feature of LMS [5, 26]. Historically, early and heterogeneous enhancement reflecting tumor vascularity has also been reported to contribute to the diagnosis [33]. Among the studies included in this meta-analysis that evaluated contrast enhancement, diagnostic criteria were inconsistent, ranging from the assessment of specific enhancement patterns [23, 24] to the simple detection of non-enhancing necrotic areas without pattern analysis [15, 16]. Consequently, its association with malignancy has not been firmly established by either qualitative or quantitative approaches [10]. Therefore, contrast administration is currently regarded most useful for distinguishing viable solid components from necrosis rather than for detailed perfusion analysis.

DWI provides objective quantitative data, where increased cellularity corresponds to restricted diffusion and low ADC values. The studies included in this meta-analysis evaluated these metrics [14, 16, 17, 19–25], generally confirming that sarcomas demonstrate marked diffusion restriction. Consistent with these findings, a large meta-analysis, by Woo et al. [11] reported that incorporating ADC values markedly improved diagnostic performance (sensitivity 94%, specificity 95%, AUC 0.94), and they identified an ADC threshold of approximately 0.904 × 10⁻³ mm²/s. This aligns with the consensus statement by Hindman et al. [10], which emphasizes that tumors with restricted diffusion (ADC value ≤ 0.9 × 10⁻³ mm²/s) are suggestive of malignancy, a threshold consistent with the cutoff proposed by Abdel Wahab et al. [16] in our reviewed studies. Recent external validations, such as by Horowitz et al. [34], have further confirmed the reproducibility of this consensus threshold. In a study comparing a radiomics model with human readers, Xie et al. [15] highlighted the clinical necessity of quantitative metrics, by reporting that standard qualitative assessment by radiologists based on conventional MRI sequences without DWI (T1WI, T2WI, and DCE), yielded low sensitivity (58.6%), because it relied heavily on typical malignant features such as frank necrosis or hemorrhage, so sarcomas lacking these specific signs were frequently misclassified as benign. In contrast, the ADC-based radiomic model achieved an AUC of 0.83 with sensitivity of 76.0%, specificity of 73.2% demonstrating the value of quantitative ADC analysis.

While individual sequences provide specific clues, the relative contribution of each sequence to the final diagnosis warrants careful consideration. Reflecting this, diagnostic strategies employing these parameters varied widely, encompassing scoring systems [18, 24], algorithm-based models [16, 23, 25], deep learning [21, 35], and radiomics [15]. Notably, given that contrast-enhanced imaging findings beyond necrosis often lack consistency, recent guidelines favor a hierarchical approach. For instance, the consensus statement by Hindman et al. [10] proposes a stepwise evaluation: initially assessing extra-uterine disease and T2 signal intensity, while relying on DWI and specific ADC cutoffs as the critical “branching points” to distinguish sarcomas from benign entities. Similarly, Rosa et al. [23] demonstrated high diagnostic accuracy using a multicenter MRI-based diagnostic algorithm integrating multiple imaging parameters, including T1WI, T2WI, DWI, and CE-T1WI. Furthermore, the integration of clinical and laboratory data is emerging as a vital strategy to enhance individualized malignancy prediction. Yamanishi et al. [25] reported promising results using a diagnostic algorithm that combines MRI findings, specifically T2 signal intensity, T2 margin characteristics, and DWI, with serum lactate dehydrogenase levels. In addition, recent studies published outside our review period have proposed updated algorithms that incorporate menopausal status, T2 signal, DWI signal, tumor margins, and an ADC cutoff value of 1.23 × 10⁻³ mm²/s to achieve high accuracy [36]. Thus, while MRI remains the cornerstone of diagnosis, integrated MRI assessment should prioritize the inclusion of DWI alongside morphological sequences. Current evidence suggests that a comprehensive approach integrating several MRI findings, especially ADC metrics, with clinical and laboratory data offers the most robust strategy for distinguishing sarcomas from benign simulators.

Although histopathology remains the definitive standard for diagnosing sarcoma, distinguishing it from leiomyoma is sometimes difficult even for experienced gynecologic pathologists. Coagulative tumor necrosis, a key criterion for malignancy, may resemble hyaline or ischemic degeneration, and interobserver agreement on its presence is only moderate (κ = 0.44) [37]. Cellular leiomyoma and STUMP further complicate interpretation, as their morphological features overlap extensively with those of sarcoma. Given that even histopathologic distinction is challenging, some degree of diagnostic difficulty on imaging is inevitable. These limitations in the reference standard highlight the need for careful imaging assessment. Clinically, misclassification has important consequences: a false-positive diagnosis may lead to unnecessary hysterectomy, whereas a false-negative result may delay appropriate surgery for a true malignancy. This diagnostic uncertainty is particularly critical in younger patients who aim to preserve fertility. Therefore, MRI evaluation should be integrated with clinical findings, laboratory data, follow-up imaging, and, when indicated, biopsy, ideally within a multidisciplinary framework to ensure individualized and oncologically appropriate management.

This review has several limitations. First, inclusion was restricted to English-language studies published from 2019 onward, which may introduce selection bias. Second, subgroup analyses were not performed, and potential sources of heterogeneity could not be formally evaluated. Third, the meta-analysis relied on study-level 2 × 2 data, preventing adjustment for covariates or harmonization of diagnostic thresholds. Fourth, most included studies were retrospective and used variable inclusion criteria, reference standards, and MRI protocols. Malignant cohorts sometimes included non-sarcomatous tumors, and benign lesions were occasionally verified by follow-up alone. Finally, disease prevalence varied widely across studies, limiting the comparability of predictive values and allowing only qualitative assessment of publication bias.

In conclusion, although uterine sarcomas are ultimately diagnosed histopathologically after surgical resection, preoperative MRI plays a crucial role in treatment planning. MRI provides substantial diagnostic value for distinguishing sarcomas from leiomyomas, despite unavoidable overlap in some cases. Standardized MRI protocols and diagnostic criteria are essential to improve diagnostic consistency and reproducibility. Future research should focus on establishing integrated diagnostic frameworks to support personalized management that balances oncologic safety with fertility preservation.

## Data Availability

No new datasets were generated or analyzed in this study. Extracted data supporting the findings of this review are available from the corresponding author upon reasonable request.
